# Nonnatural amino acid incorporation into the methionine 214 position of the metzincin *Pseudomonas aeruginosa *alkaline protease

**DOI:** 10.1186/1471-2091-6-21

**Published:** 2005-10-12

**Authors:** Paula Walasek, John F Honek

**Affiliations:** 1 Department of Chemistry, University of Waterloo, 200 University Avenue, Waterloo, Ontario, Canada N2L 3G1

## Abstract

**Background:**

The alkaline protease from *Pseudomonas aeruginosa *(AprA) is a member of the metzincin superfamily of metalloendoproteases. A key feature of these proteases is a conserved methionine-containing 1,4-tight β turn at the base of the active site zinc binding region.

**Results:**

To explore the invariant methionine position in this class of protease, incorporation of a nonnatural fluorinated methionine, L-difluoromethionine (DFM), into this site was accomplished. Although overproduction of the N-terminal catalytic fragment of AprA resulted in protein aggregates which could not be resolved, successful heterologous production of the entire AprA was accomplished in the presence and absence of the nonnatural amino acid. DFM incorporation was found to only slightly alter the enzyme kinetics of AprA. In addition, differential scanning calorimetry indicated no significant alteration in the thermal stability of the modified enzyme.

**Conclusion:**

Although invariant in all metzincin proteases, the methionine 214 position in AprA can be successfully replaced by the nonnatural amino acid DFM resulting in little effect on protein structure and function. This study indicates that the increased size of the methyl group by the introduction of two fluorines is still sufficiently non-sterically demanding, and bodes well for the application of DFM to biophysical studies of protein structure and function in this class of protease.

## Background

The metzincin superfamily of proteases includes the matrixins, astacins, reprolysins, snapalysins, leishmanolysins and serralysins [1,2]. These zinc-dependent endoproteases may act on a large number of substrates, or they may be highly specific, targeting only one or a few proteins or oligopeptides. There are over 700 different enzymes that have been classified as metzincins and they are produced by organisms ranging from bacteria to plants and animals [3]. As diverse as the members of this superfamily are, surprisingly they all share a structurally similar catalytic site which includes a common zinc binding motif and an absolutely conserved methionine-containing 1,4-tight β turn, termed the "Met-turn" in their active sites [4].

**Figure 1:**
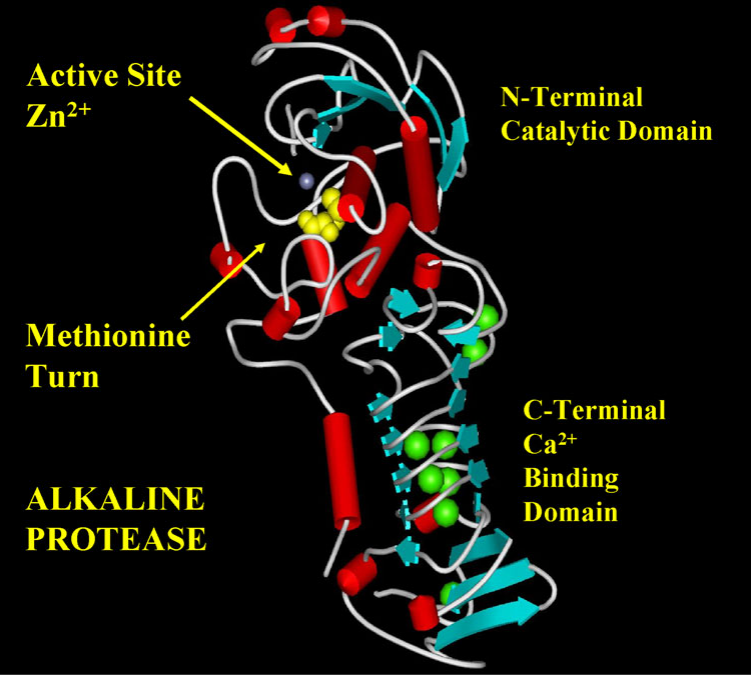
Ribbon representation of alkaline protease from *P. aeruginosa *(AprA). The active site zinc ion is shown in blue and the conserved methionine in yellow spacefill just below the zinc. Calcium ions in the C-terminal β-roll domain are green. Figure generated using WebLab Viewer Pro 3.7 using PDB entry 1KAP [15].

Many of the metzincin proteases are of medical significance. The matrixins, or matrix metalloproteinases (MMPs) include the collagenases, gelatinases and stromelysins [5,6]. Members of this subfamily not only degrade extracellular matrix for tissue remodeling and maturation, but they have also been found to release and activate several growth factors and modulate chemotactic signals [5]. In disease processes, however, they have been implicated in promoting tumour invasion and metastasis by degradation of matrix barriers, exacerbating periodontal disease, and destroying aggrecan and collagen in rheumatoid arthritis. For this reason, much interest has arisen in mechanistic and inhibitor studies of MMPs [7-9]. The astacins comprise a subfamily of enzymes involved in digestion, developmental regulation, and peptide processing. The reprolysins or adamalysins are mainly responsible for the hemorrhagic effects and tissue necrosis typical of snake bites [2]. These effects are caused mainly by destruction of the extra-cellular matrix surrounding capillaries in conjunction with the inhibition of platelet aggregation by disintegrins. The N-terminal catalytic domains of members of this family are usually zinc and calcium dependent, while the C-terminal portion may be disintegrin, cysteine-rich, or lectin type domains with hemorrhagic or cell adhesion properties. Mammalian adamalysins play important roles in reproduction, myogenesis and cytodifferentiation as well as in disorders such as asthma, cardiac hypertrophy, and endotoxic shock [10-13]. The snapalysins are secreted proteases from several *Streptomyces *organisms and the membrane-bound leishmanolysins originate from a number of protozoan parasites. The serralysins, of which the alkaline protease from *P. aeruginosa *(AprA) is a member, are secreted by several pathogenic, gram-negative bacterial species [14]. They are, on average, 50 kDa proteins consisting of 2 domains; the C-terminal domain binds up to eight calcium ions in a mainly structural role, but also contains the secretion signal. The catalytic site is located in a cleft on the N-terminal domain (Figure 1)[15]. The physiologic substrate(s) of these enzymes are not known with certainty, but are thought to be proteins of the extracellular matrix and possibly immune response elements such as interferon gamma and complement proteins [16,17].

To add a further dimension to the techniques applied to the analysis of protein structure and function, the incorporation of nonnatural amino acids into proteins is currently of great interest. One aspect of this approach is the incorporation of amino acids containing fluorine which would be applicable to not only probing subtle steric interactions in proteins (Van der Waals' radius of F is 1.47 Å vs. 1.20 Å for H), but also in applying ^19^F spectroscopy to this area [18,19]. In order to probe the methionine position in this class of proteins (specifically AprA), the fluorinated analogue, S-difluoromethyl-L-homocysteine (L-difluoromethionine, (DFM)), was utilized to replace the methionine in the Met-turn and to determine its effects on catalytic activity and thermal stability.

## Results

### N-terminal catalytic fragment

Attempts to overproduce the N-terminal catalytic fragment of AprA which consisted of residues T40 to N258 of the 479 residue protein led to the production of aggregates in spite of attempts to increase soluble protein expression by control of temperature, isopropyl-β-D-thiogalactopyranoside (IPTG) concentration, co-expression of folding chaperones (GroES and GroEL) as well as secretion and fusion to a NusA solubility tag [20-23]. In several attempts at resolubilisation of these inclusion bodies (see Methods for details), semi-soluble aggregates of the truncated AprA were detected by gel filtration chromatography (Superose-12) having molecular weights higher than the exclusion limit of the resin (>2 × 10^6 ^Da). Due to the difficulty of isolating and handling sufficient quantities of this truncated version of AprA, attention was then focused on overproduction of the full-length mature form of AprA which corresponds to residues G10 to V479.

### Mature full-length AprA and DFM incorporation

**Figure 2:**
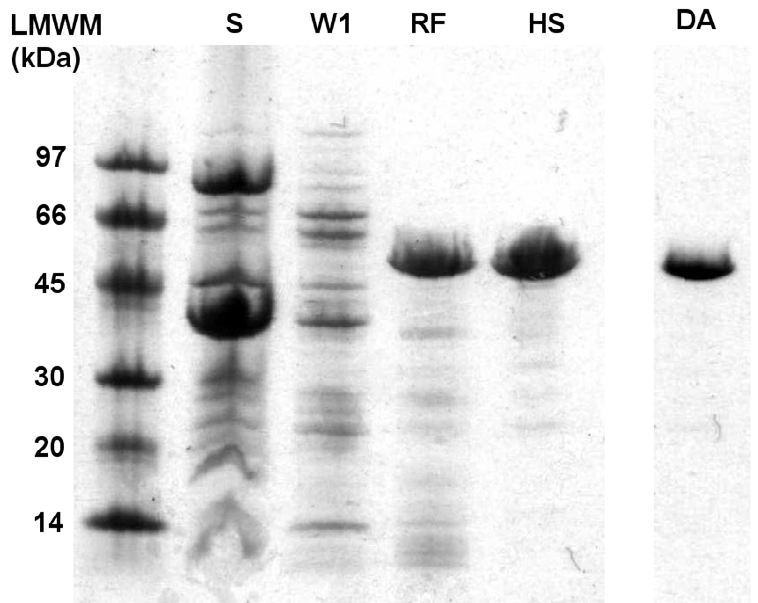
SDS-PAGE gel summary of the refolding of mature full-length AprA. S represents the supernatant of the crude lysate, W1 is the solution from the first wash of the inclusion body pellet, RF is the refolded protein and HS is the refolded protein after the high salt wash and DA is the refolded DFM-AprA.

**Figure 3:**
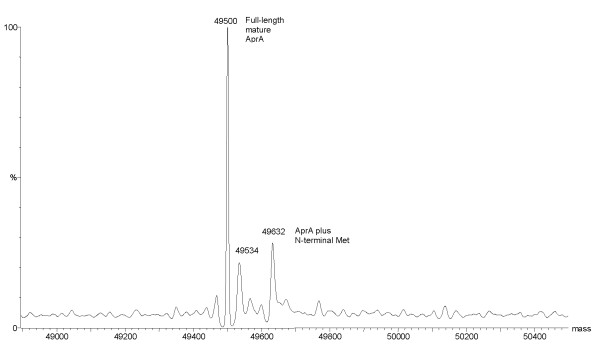
Positive ion mode electrospray mass spectrum of the refolded, full-length, mature AprA. The major peak in the spectrum (49 500 Da) corresponds exactly to the recombinant protein without the initiator methionine. The peak at 49 534 Da may represent a calcium adduct. The peak at 49 632 Da corresponds to AprA retaining the N-terminal methionine.

**Figure 4:**
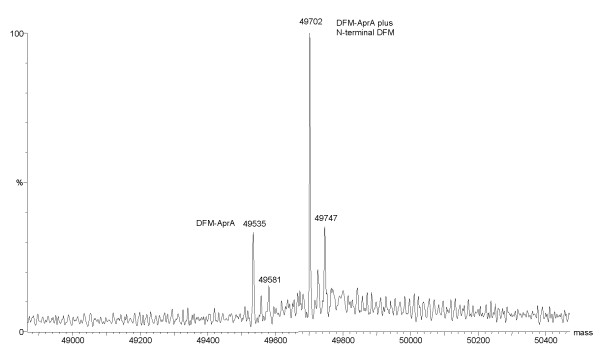
Positive ion mode electrospray mass spectrum of DFM-AprA. The calculated molecular weight of the labelled protein with the initiating residue was 49 702 Da, and without, 49 535 Da. Possible sodium adducts (2Na^+^) are seen for both species at 49 747 Da and 49 581 Da.

The gene for the full-length mature form of AprA [24-26] which corresponds to residues G10 to V479 was isolated on to pET22b and overexpressed in *E. coli *BL21 (λDE3), resulting in inclusion body formation. Isolation of the protein aggregates followed by solubilisation and refolding produced an active, stable and soluble enzyme of 49 500 Da, according to mass spectrometric analysis (Figure 2 and 3). L-DFM was synthesized as previously described [27,28] and incorporated into AprA using the methionine auxotrophic *E. coli *strain, B834 (λDE3). The resulting inclusion bodies were refolded in an identical manner as the non-labelled protein, and the expected 49 535 Da protein, as determined by electrospray mass spectrometry (ESMS), was isolated (Figure 2 and 4).

### Kinetic analysis and differential scanning calorimetry

Utilizing a previously reported spectrophotometric method for kinetic analysis of AprA, the mature, full-length recombinant protein showed similar kinetic properties for hydrolysis of Z-Arg-Arg-p-nitroanalide to those reported for the mature wild-type protein, isolated directly from *P. aeruginosa *growth media [29]. The k_cat _values were determined to be 1.6 ± 0.1 min^-1 ^versus 0.6 ± 0.1 min^-1 ^and the K_m _values were 12.6 ± 2.4 μM vs. 6 ± 1 μM for the enzyme produced by the cognate organism and the recombinant protein respectively.

Slightly improved assay conditions were found by increasing the amount of enzyme from 25 μg per assay to 100 μg in order to improve sensitivity, and by replacing the assay buffer from 5 mM TRIS to 20 mM HEPES to avoid possible metal binding effects of the TRIS buffer. The kinetic parameters of non-labelled and DFM-labelled AprA proteins were compared under the new conditions and found to be similar. The K_m _was not significantly altered (15.4 ± 0.9 μM for the unlabelled protein vs. 14 ± 2 μM for the DFM-incorporated), and the k_cat _was only slightly affected by DFM incorporation, showing a decrease from 0.57 ± 0.02 min^-1 ^to 0.44 ± 0.02 min^-1^.

**Figure 5:**
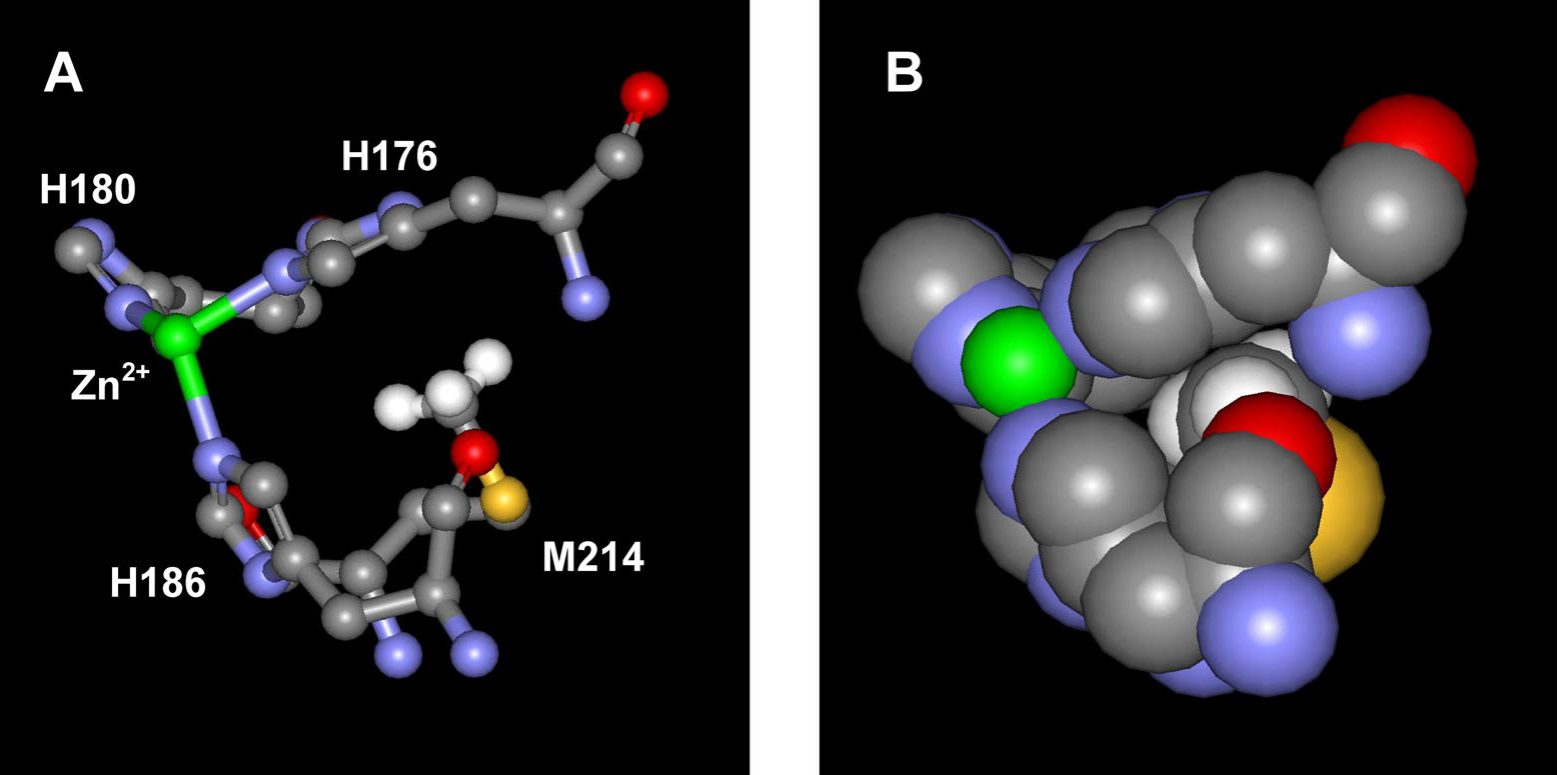
Active site of AprA showing the environment of the conserved methionine (M214). A: The three histidine ligands (labelled) are shown along with the zinc ion (in green). The spacefill representation (B) shows the closely packed arrangement around the methionine residue, as well as its proximity to the catalytic zinc and the three histidine residues. Protons (white) were added to the M214 methyl group for clarity. Based on structure from PDB entry 1KAP [15].

**Figure 6:**
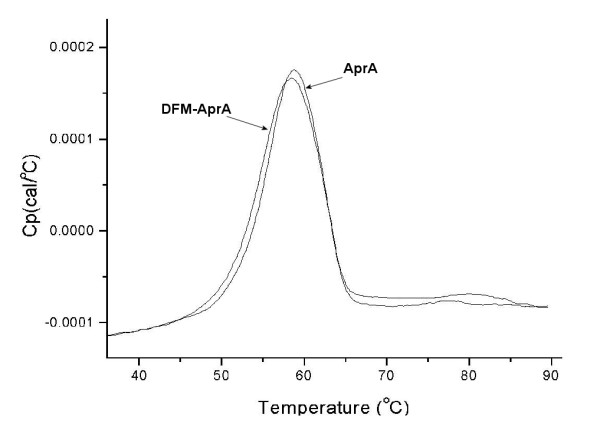
Differential scanning calorimetry data of both the unlabelled AprA and the DFM-incorporated AprA. All scans were performed at 1°C per minute in 20 mM HEPES (pH 7.8) and buffer scans were subtracted from sample runs.

Due to the proximity of the Met214 residue to the metal ligands which ligate the zinc ion (Figure 5), the effect of substitution of this Met with the larger DFM residue on thermal denaturation was investigated. Replicate differential scanning calorimetry experiments were performed on both the mature full-length AprA and the DFM-labelled mature full-length AprA (DFM-AprA) in 20 mM HEPES (pH 7.8). The T_max _for unlabelled and DFM-labelled AprA were determined to be 58.7 ± 0.1°C and 58.6 ± 0.2°C respectively (Figure 6).

### ^19^F NMR spectroscopy

**Figure 7:**
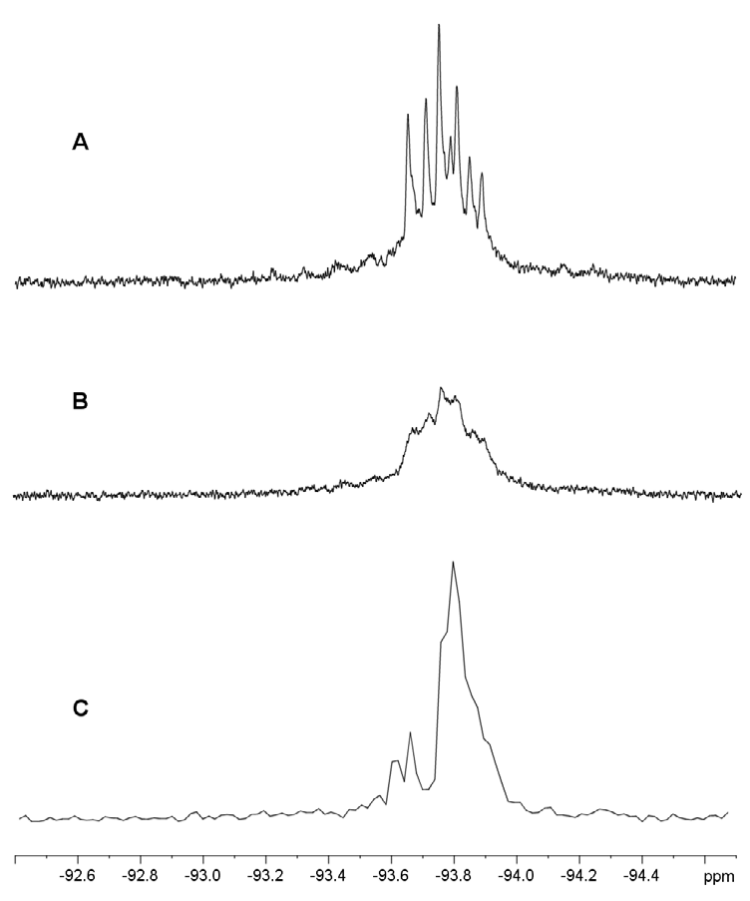
^19^F NMR spectra of DFM-labelled AprA. The 564.7 MHz ^19^F NMR spectrum of DFM-labelled AprA in D_2_O with approximately 40 mM HEPES (A) and with the addition of 2 mM Gd(EDTA)^- ^(B). Proton-decoupled spectrum of DFM-labelled AprA (C).

DFM-labelled mature full-length AprA was analyzed by ^19^F nuclear magnetic resonance (^19^F NMR) spectroscopy without proton decoupling to produce a complex set of ^19^F resonance multiplets centered at approximately -93.8 ppm. Paramagnetic line broadening with addition of gadolinium(III)-N,N,N',N' -ethylenediaminetetraacetic acid ((GdEDTA)^-^) complex resulted in line broadening of all ^19^F resonances. Proton-decoupled ^19^F NMR experiments resulted in collapse of the ^19^F multiplets, however complete resolution of the N-terminal DFM from the internal (DFM214) ^19^F resonances was still incomplete at this field strength (564.5 MHz) (Figure 7).

## Discussion

AprA is a member of the metzincin superfamily of metalloproteases and is one of several proteases secreted from *Pseudomonas aeruginosa *into tissue of infected patients [16,30,31] (Figure 1). It appears that a combination of AprA, elastase (LasB) and LasA protease and protease IV work in concert to produce tissue invasion [32,33]. AprA is biosynthesized in *P. aeruginosa *as a 479 residue polypeptide which is secreted via a C-terminal signal sequence directly into the extra cellular medium where a 9 amino acid N-terminal inhibitory propeptide is autocatalytically removed [26]. A critical aspect to the structure-function of AprA and the metzincins in general is the active-site arrangement of the metal ligands and the still incompletely understood methionine-containing 1,4-tight β turn at the base of the active site present in all members of the metzincin superfamily. Although much is known concerning the cellular biochemistry of a number of metzincins, detailed knowledge of the function of the components of the active site is still lacking. For example, methionine residues in proteins are less frequent in occurrence than many other residues (third least abundant after tryptophan and cysteine [34]) and it can frequently be replaced with other hydrophobic amino acids such as leucine [35,36]. However the surprising persistent presence of methionine in over 700 members of this class of protease has been noted[3,4,37,38]. Attempts to define the contribution that this seemingly quintessential amino acid plays in this class of enzyme have indicated that, depending upon the specific superfamily member studied, some flexibility in replacement may be possible, at least *in vitro*. For example, in the case of MMP-2 (gelatinase A), enzymes with replacements of the active site Met with either Leu or Ser have only minor perturbations in catalytic activity [39]. In the case of protease C from *Erwinia chrysanthemi*, replacement of the active site methionine (Met226) by leucine, alanine or isoleucine resulted in enzyme mutants sufficiently stable to be isolated, but this was not so with M226H, M226S or M226N mutations. However the enzyme kinetic properties of the M226L, M226A and M226I mutants were compromised and crystallographic studies indicated some alteration of the zinc binding histidines which might explain the effects of these mutations on the kinetic properties of the enzyme [40].

Substitution of the active site Met by selenomethionine (SeMet) in MMP-8 (human neutrophil collagenase) was also found to be somewhat perturbing. In the catalytic domain of MMP-8, SeMet appeared to decrease the protein's resistance to urea denaturation [41]. This loss of stability was accompanied by a considerable decrease in activity and an increase in K_m _towards the substrate (7-methoxycoumarin-4-yl)acetyl-Pro-Leu-Gly-Leu-(3- [2,4-dinitrophenyl]-L- 2,3-diaminopropionyl)-Ala-Arg-NH_2_. In a previous study using the substrate (dinitrophenyl-Pro-Leu-Gly-Leu-Trp-Ala-D-Arg-NH_2_), however, k_cat _and K_m _values were unaffected by SeMet replacement in MMP-8 [42,43].

Evidently additional investigations into the properties of the metzincins through modification of the methionine position may be of importance. However the usual ensemble of potential replacements of amino acid residues through site-directed mutagenesis is limited in general to replacement with the other nineteen amino acids. In order to explore the effects of alterations of the critical methionine in the metzincin class of proteases, the introduction of a sterically and electronically subtle methionine analogue DFM, was the focus of the present study. For these studies the alkaline protease from *Pseudomonas aeruginosa *was initially investigated as it is representative of the metzincin family of proteases, is important in tissue invasion by this pathogen and has only two methionines in its structure, the N-terminal methionine and the Met-turn methionine, M214. The presence of only two methionine residues in this metzincin (and only the active site methionine if a mature secreted AprA is produced) facilitates bioincorporation of DFM into the protein without the use of complex cell free in-vitro translation system approaches [44-46]. As well, this choice of target protein for a preliminary study avoids complications with additional bioincorporation of the analogue into other methionine positions which may be present in other members of the metzincin superfamily.

DFM is a subtle derivative of methionine that has been shown to be useful as a ^19^F-NMR probe in proteins [28,47]. Unlike leucine and isoleucine, this unbranched analogue may only produce a moderate increase in the steric properties of the methyl group due to the incorporation of two fluorine atoms. In addition, fluorination could profoundly affect the electron density of the sulfur atom [27]. As can be seen in Figure 5, the thiomethyl group of M214 is in close proximity to H176, H180, and H186 and may contribute to the proper positioning of these metal ligands. Hence it was of interest to explore the effects of this nonnatural amino acid on the catalytic activity and thermal stability of the modified protein by introduction of L-difluoromethionine into the critical methionine position in the Met-turn.

Previous studies on other metzincins such as stromelysin, MMP-8, and astacin have shown that the truncated enzymes containing only the catalytic domain of these enzymes could be expressed and were sufficiently stable to be studied [48-50]. In the current study, initial attempts were therefore focused on the overproduction of the smaller N-terminal catalytic domain of AprA (T40 to N258). Although evidence for cellular production of this fragment was observed, almost complete aggregation of this fragment occurred under a variety of experimental conditions designed for its control (Results). Previous reports by Guzzo and co-workers have shown that even minimal recombinant expression (1 μg/mL) of soluble, intact AprA protein required the co-expression of the entire secretion apparatus (AprD, AprE, AprF) [51]. When the structural gene for AprA was expressed alone, however, intracellular accumulation of AprA was barely detectable, most likely due to rapid degradation inside the cell [52]. In other reports, significant quantities of the enzyme could be found intracellularly, but only in the form of inclusion bodies, which are known to be highly resistant to degradation [24,53,54].

However successful production of mature full length (G10 to V479) AprA, was accomplished without the co-expression of the entire secretion apparatus. Although insoluble protein aggregates were produced, it was possible to resolubilise and refold the overexpressed protein, yielding approximately 40 mg of high purity enzyme per 1 L of culture (Figure 2). The ESMS analysis revealed that although the majority of the protein isolated from this protocol had M_r _= 49 500 Da (full length mature AprA, G10-V479), a small quantity of inseparable enzyme with an M_r _= 49 632 Da had maintained its N-terminal methionine (Met-G10-V479) from the cloning procedure (Figure 3). Kinetic analysis of this recombinant mature AprA compared favourably with values previously reported [29] for the wild type AprA secreted by *P. aeruginosa *(k_cat_/K_m _of 2140 ± 540 M^-1^s^-1 ^and 1800 ± 300 M^-1^s^-1 ^for wild type and recombinant enzymes respectively).

An almost 100% incorporation level of the fluorinated probe into AprA was readily accomplished by induction of protein expression in minimal media supplemented with 2 mM DFM. The labelled protein was also expressed as intracellular inclusion bodies which were resolubilised and refolded using the same protocol as the unlabelled recombinant protein, yielding approximately 16 mg of nearly homogeneous protein per 1 L of culture (Figure 2). The ESMS analysis indicated that, unlike the unlabelled recombinant AprA, a majority of the DFM incorporated protein maintained the initiating residue (Mr = 49 702 Da), while this DFM was cleaved in only about one quarter of the sample (Mr = 49 535 Da) (Figure 4). The interesting observation that removal of the N-terminal DFM residue occurs to a lesser extent compared to methionine in this position indicates that the processing of fluorinated methionines by methionine aminopeptidase is a less efficient process. Related to this observation is a recent report by Budisa and coworkers indicating the lack of removal of trifluoromethionine from the N-terminus of a mutant green fluorescent protein[55]. It may be that the increased steric size of the fluorinated methyl groups in these analogues alters the correct positioning of the peptide bond of the substrate in the active site of the methionine aminopeptidase which could reduce hydrolytic efficiency by this enzyme. In any event, the presence of the N-terminal methionine (or DFM) is not expected to affect catalytic activity or thermal stability of the protein as the N-terminus is physically remote from the active site. The kinetic characteristics of the protein mixture with and without incorporated DFM were determined under the revised conditions described in the methods and results sections. The K_m _was not significantly altered by replacement with DFM, the V_max _and therefore k_cat _were only slightly impaired, resulting in a decrease in k_cat_/K_m _from 620 ± 30 M^-1^s^-1 ^to 530 ± 50 M^-1^s^-1^.

In order to compare the effects of DFM on thermal stability of the protein, preliminary differential scanning calorimetry studies were undertaken on AprA and DFM-AprA. Under the conditions utilized in these experiments, the thermal denaturations of AprA and DFM-AprA were found to be irreversible. As shown in Figure 6, the thermal stability in 20 mM HEPES (pH 7.8) was not significantly altered upon fluorination of the Met-turn methionine. The T_max _for unlabelled and DFM-labelled AprA were determined to be 58.7 ± 0.1°C and 58.6 ± 0.2°C respectively. This would indicate that the introduction of two fluorine atoms into the methyl group, which is in close contact to the critical histidines in the active site, can be accommodated by the protein such that the catalytic properties and thermal stability of AprA are not drastically perturbed.

Due to the lack of background ^19^F resonances in most biological systems, the 100% natural abundance of the ^19^F isotope and its sensitivity to NMR detection of 83 % to that of ^1^H, the application of ^19^F spectroscopy to studies in protein structure and function has been an important biophysical technique that has been shown to give critical information on the environment surrounding the fluorine nucleus [19,56-58]. Previous studies on DFM incorporation into the transglycosylase of bacteriophage λ and the leucine-isoleucine-valine (LIV) binding protein from *E. coli*, have detected separate sets of resonances for each DFM residue present in each protein and their presence has been useful in detection of ligand binding [28,47]. As the two fluorine atoms in DFM are diastereotopic due to the presence of the chiral centre of the α-carbon of methionine, complex ^19^F resonances can result. In fact, it has been shown that a complex octet of ^19^F resonances is observed when a DFM residue is located in a conformationally restricted protein environment [28]. It was therefore of interest to investigate the ^19^F NMR spectroscopy of DFM-AprA. The ^1^H-coupled ^19^F NMR spectrum of DFM-AprA (Figure 7A) is a composite of the ^19^F resonances not only from the active site DFM at position 214, but also a contribution from the presence of protein molecules still maintaining the N-terminal DFM residue. Interestingly the two resonances overlap in the 564 MHz spectrum. It has previously been shown that application of paramagnetic line broadening agents, such as Gd(EDTA)^- ^will broaden the ^19^F NMR resonances from fluorine nuclei in a protein in a distance-dependent manner and can serve to indicate which ^19^F resonances originate from more solution exposed residues [56,58]. To possibly distinguish the signal contributions from each DFM, paramagnetic line broadening experiments utilizing Gd(EDTA)^- ^were undertaken. These experiments (Figure 7B) indicated that both DFM residues (DFM1 and DFM214) were equally susceptible to the line-broadening agent, indicating that close approach to the active site DFM by Gd(EDTA)^- ^was also possible in solution. Based on the crystal structure of AprA [15,59], the active site is open to solvent and could be expected to reasonably accommodate the rather large Gd(EDTA)^- ^molecule resulting in little discrimination between the two DFM residues. Although proton decoupling simplified the ^19^F spectrum (Figure 7C), the resonances from the two DFM residues were still unresolved; the complexity of which may also result from contributions of the restricted environment of the active site DFM. Attempts to remove the N-terminal DFM with recombinant *E. coli *methionine aminopeptidase [60] under a variety of conditions resulted in complex mixtures due to proteolytic degradation by AprA. Additional attempts to produce a variant of AprA lacking the N-terminal DFM residue by introduction of the wild-type N-terminal self-cleaving propeptide sequence or a thrombin cleavage site resulted in extremely poor isolated yields of these forms of the protease. Further studies will be focused on the application of this NMR probe to investigating conformational changes in the active site upon ligand and inhibitor binding.

## Conclusion

The ^19^F biophysical probe, DFM, was successfully incorporated into the critical Met-turn of AprA and resulted in minimal catalytic or structural alteration of the enzyme. These findings are encouraging for the further application of DFM as a subtle probe for the investigation of the metzincin family of proteases.

## Methods

### Materials

Restriction endonucleases were purchased from New England BioLabs (Pickering, ON). The Expand™ and *Pwo *polymerase kits as well as the alkaline phosphatase (from calf intestine) were Roche (Laval, QC) products and the *Taq *polymerase was from Qiagen (Mississauga, ON). The thrombin (from bovine serum) was purchased from Amersham Biosciences (Baie d'Urfé, QC). The peptide substrate, Z-Arg-Arg-pNA, was purchased from Bachem (King of Prussia, PA). The VP-DSC differential scanning microcalorimeter and Origin™ software (Version 5.0) were from MicroCal™ (Northampton, MA). ^19^F NMR data were collected on Bruker Avance (Milton, ON) NMR spectrometers (500 and 600 MHz). Unless otherwise noted, all reagents and chemicals were of the highest commercial grade available and were used without further purification. L-Difluoromethionine was prepared by a previously reported method [28].

### Cloning and bacterial expression

The genes of interest were amplified by PCR and digested using the primers and restriction endonucleases listed in Table 1. With the exception of pNusAPA, all PCR products were ligated in to appropriately digested pET22b(+) (Novagen Inc). The NusA fusion protein was produced from a pET43.1b(+) based vector (Novagen Inc). New constructs were confirmed by DNA sequencing (Mobix, Inc) in both directions and the protein products were analyzed by electrospray mass spectrometry (positive ionisation mode).

**Table 1:** Primers and endonucleases used in the cloning of the various constructs. Restriction endonuclease cut sites are underlined and the regions of the primers homologous to the gene of interest are in bold.

Construct	Primers	Restriction endonucleases	Protein product
pAPA'	N-terminal: 5' CCA GAA TTC CAT ATG ** ACC GTC GAC CAG GCG GCG GAG C **3' C-terminal: 5' CCA GGA TCC TTA **GTT GGC CCC GTA GAG CTT CTG G **3'	*Nde*I *Bam*HI	AprA catalytic fragment (APA')
pPelAPA'	N-terminal: 5' CCG AAT TCC ATG G **CC GTC GCC GTC GAC CAG GCG GCG GAG C **3' C-terminal: Same as pAPA'	*Nco*I	APA' with pelB secretion signal
pNusAPA'	N-terminal: 5' **G GGC GGG GAC GAA TTG GTC AAT GGC **3' C-terminal: Same as pAPA'	Blunt end ligation at N-terminus: no digestion required	APA' with NusA solubility tag and His_6 _tag
pWApr	N-termnal: 5' CCA GAA TTC CAT ATG **GGT CGT AGC GAT GCG TAT ACC **3' C-terminal: 5' CCA GGA TCC **TCA GAC GAC GAT GTC GGC CTG G **3'	*Nde*I *Bam*HI	Mature AprA without pro-peptide

Unlabelled proteins were produced in the *E. coli *strain BL21 (λDE3), while strain B834 was used for DFM incorporation. A 10 mL starter culture (LB broth with 50 mg/mL carbenicillin) was inoculated from frozen stock and incubated overnight at 37°C with shaking. For the production of unlabelled proteins, the entire starter was used to inoculate 1 L of LB broth (with 50 mg/mL ampicillin) and the culture was grown to an OD_600 _of 0.6 to 0.8. IPTG was added directly to a final concentration of 1 mM and the culture was incubated at 37°C with shaking for an additional 4 hours. A similar procedure to that described by Vaughan *et al. *[28] for DFM incorporation into phage lysozyme was used to produce the L-DFM labelled AprA protein. In brief, a 10 mL starter culture of *E. coli *strain B834 harbouring plasmid pWApr was used to inoculate 1 L of M9 buffer (pH 7.2: 47.6 mM Na_2_HPO_4_, 22 mM KH_2_PO_4_, 8.5 mM NaCl, 18.7 mM NH_4_Cl, 2 mM MgSO_4_, 0.1 mM CaCl_2_) supplemented with 0.4 % glucose, 50 μg/mL ampicillin, and 0.1 mM L-Met. The culture was grown at 37°C with shaking to an OD_600 _of approximately 0.7 and then harvested by centrifugation for 10 minutes at 5000 *g*. The cells were washed in M9 buffer and then resuspended in M9 supplemented with 0.4 % glucose and 50 μg/mL ampicillin. After incubating at 37°C with shaking for 30 minutes, 2 mM L-DFM and 1 mM IPTG were added, and the culture was incubated (37°C with shaking) for a further 8 hours.

### Refolding of the N-terminal catalytic domain

The expressed N-terminal catalytic domain segment that was initially produced was extracted and solubilised in much the same manner as the full-length protein described below. However, several variations to the refolding buffer were made in attempts to recover the soluble, active catalytic fragment. A range of pH conditions from 7.2 to 8.0 was used. ZnSO_4 _was added to the refolding buffer with concentrations ranging from 0.1 μM to 0.1 mM. Glycerol (20 %) was also added to the refolding buffer and the L-arginine content was varied from 0.8 M down to 0.2 M. The arginine was replaced completely in several other attempts which instead involved 1 % Brij-35, 1 M non-detergent sulfobetaine 201 (NDSB-201), or 1 M KCl with 20 % glycerol. Finally, the dialysis buffer was also varied to include 10–20 % glycerol, 0.05 % Brij-35 or 0.05 % Triton-X 100.

### Solubilisation and refolding of the full-length AprA protein

Induced *E. coli *BL21 (λDE3) cells harbouring plasmid pWApr were harvested in a 10 min spin at 5000 *g*, washed in M9 buffer (47.6 mM Na_2_HPO_4_, 22 mM KH_2_PO_4_, 8.5 mM NaCl, 18.7 mM NH_4_Cl, 2 mM MgSO_4_, 0.1 mM CaCl_2_) then resuspended in lysis buffer (50 mM TRIS (pH 7.8), 1 mM EDTA, 0.1 % Triton-X-100) and disrupted by eight 20 second rounds of continuous sonication with cooling. The crude cell lysate was centrifuged for 10 minutes at 6000 *g*, and the pellet was washed twice in lysis buffer and then 3 times in lysis buffer without Triton.

The resulting inclusion body pellet was dissolved in a small amount of solubilisation buffer (50 mM TRIS, pH 7.8, 1 mM EDTA, 6 M guanidine hydrochloride) and incubated at 37°C for 1 hour. The solution was then centrifuged for 10 minutes at 13000 *g *and a 4 μL aliquot was diluted to up to 1 mL in solubilisation buffer for an absorbance measurement at 280 nm. Protein concentration was estimated using a predicted extinction coefficient of 67 990 M^-1^cm^-1 ^calculated by the ProtParam tool in ExPASy [61]. Additional solubilisation buffer was used to dilute the protein solution to 10 mg/mL. This solution was then passed through a 0.22 μm syringe filter and added dropwise with stirring to ice cold refolding buffer (50 mM TRIS, pH 7.8, 1 mM CaCl_2_, 0.8 M L-arginine), resulting in a 100-fold dilution (final refolding protein concentration: 0.1 mg/mL). After an overnight incubation with stirring at 4°C, the protein was dialysed against two changes of buffer with metals (20 mM HEPES, pH 7.8, 1 mM CaCl_2_, 0.1 mM ZnSO_4_), then one without CaCl_2 _or ZnSO_4_, but with 300 mM NaCl. Finally, the protein was concentrated using an Amicon pressure filtration device with a 10 kDa cut-off filter and the high salt buffer exchanged to 20 mM HEPES (pH 7.8).

### Mass spectrometry

Electrospray mass spectrometry of purified protein samples was carried out on a Micromass Q-TOF Global Ultima mass spectrometer in positive ion mode. Spectra were analyzed using the MaxEnt1 algorithm in the MassLynx (V4.0, Micromass Limited/Waters, Milford, Massachusetts) software. Buffer salts were removed from protein samples using a Nanosep microcentrifuge concentrator with a 10 kDa cut-off. Once in Milli-Q (Waters Corp.) purified water, samples were diluted with a solution of 50 % acetonitrile and 0.1 % formic acid.

### Activity assay

Initial determinations of kinetic parameters using the substrate Z-Arg-Arg-p-nitro-analide were carried out under the conditions described by Louis *et al. *for wild type alkaline protease [29], using substrate concentrations ranging from 3 μM to 200 μM. The rate of p-nitroanaline released was calculated using the extinction coefficient of 8800 M^-1^cm^-1 ^at 410 nm [29]. In order to avoid possible metal chelating effects of the TRIS buffer and to increase assay sensitivity, 20 mM HEPES buffer was used in place of the 5 mM TRIS, and 100 μg of protein was used for each assay. Kinetic parameters, K_m _and V_max_, were determined by non-linear regression using the GraFit5™ software (Erithacus Software Limited).

### Differential scanning calorimetry

A protein concentration of 0.5 mg/mL was used for each melting temperature determination. Initially, 50 mM TRIS (pH 7.8) was used as the buffer for these experiments, however, this was eventually replaced by 20 mM HEPES. Therefore, protein samples in 20 mM HEPES (pH 7.8) and reference buffer (20 mM HEPES, pH 7.8) were degassed under vacuum just prior to analysis. Scanning was performed continuously between 15 and 90°C at a rate of 1°C per minute with a 1 minute hold in between each up and down scan. An initial buffer-buffer scan was performed in order to establish a thermal history for the system. The subsequent buffer-buffer scan was then used as a reference. Once the temperature was below 35°C again, the protein solution was loaded into the sample cell and scanned several times.

Data were analysed using the Origin5.0(™) software in the following manner. The reference buffer-buffer scan was subtracted from the subsequent protein scan, the data were normalised for protein concentration, and the temperature (T_max_) at the resulting peak was determined. Plots of heat capacity, *Cp*, versus temperature were then plotted. (T_max _may be defined as the apparent temperature of maximum excess specific heat absorption [62,63]. The T_max _values for both the mature wild type AprA and the DFM-incorporated proteins were determined in triplicate, averaged and the standard deviation determined.

### ^19^F NMR spectroscopy of DFM-labelled AprA

Protein was first dialysed against 5 mM HEPES (pH 7.8) and then lyophilised and stored at -20°C until needed. Approximately 4 mg of lyophilised protein was made up in 500 μL of D_2_O for analysis. ^19^F NMR (564.5 MHz) data were collected using a Bruker Avance 600 MHz spectrometer fitted with a 5 mm dual ^19^F/^1^H probehead tuned to ^19^F. Standard parameters were11 261 Hz sweep width, 1.45 second acquisition time and a 0.5 second relaxation delay. A 1 Hz line broadening was applied. Spectra were recorded at 277 K and referenced to an external sample of trifluoroacetic acid (set at-76.53 ppm) or a solution of 0.05 % trifluorotoluene in CDCl_3 _(set at -63 ppm) as external references. Gd(EDTA)^- ^line broadening experiments were realized by adding 13 μL and later 26 μL of a stock solution of Gd(EDTA)^- ^(80 mM GdCl_3_, 500 mM EDTA, pH 7.1 with NaOH) to the sample.

## Authors' contributions

PW carried out the cloning and protein isolation and characterization. Both authors drafted the manuscript. JFH conceived of the study, and participated in its design and coordination. Both authors read and approved the final manuscript.
